# Keratoacanthomas have an immunosuppressive cytokine environment of increased IL-10 and decreased GM-CSF compared to squamous cell carcinomas

**DOI:** 10.1038/sj.bjc.6690552

**Published:** 1999-07

**Authors:** M A Lowes, G A Bishop, B E Cooke, R StC Barnetson, G M Halliday

**Affiliations:** Department of Medicine (Dermatology), University of Sydney at Royal Prince Alfred Hospital, Room W210, Blackburn Building D06, Sydney, NSW 2006, Australia; Liver Immunobiology Unit, Centenary Institute of Cancer Medicine and Cell Biology, Sydney, NSW, Australia; Anatomical Pathology, Prince of Wales Hospital, Sydney, NSW, Australia

**Keywords:** skin cancer, tumour regression, tumour immunology, tumour development

## Abstract

To investigate the relationship between keratoacanthoma (KA) and squamous cell carcinoma (SCC), cytokine mRNA in 12 KA and eight SCC were compared. Normal skin was also studied. Reverse transcription polymerase chain reaction (RT-PCR) was used to quantitate mRNA in each sample utilizing DNA standards. Glyceraldehyde-3-phosphate dehydrogenase (GAPDH) was used as an internal control, and CD3δ as an indication of the T-cell infiltrate. KAs showed a significant increase in interleukin (IL)-10, and a decrease in granulocyte macrophage colony-stimulating factor (GM-CSF) mRNA compared to SCCs. CD3δ mRNA was also increased in the KAs. There was no difference between KAs and SCCs in expression of lymphotoxin-α, IL-2, interferon-γ (IFN-γ), IL-13, transforming growth factor-β (TGF-β), or the pro-inflammatory cytokines IL-8 or tumour necrosis factor-α (TNF-α). These results indicate that KAs spontaneously resolve in an immunosuppressive environment. KAs grow rapidly over a period of weeks and then involute. It is possible that a suppressed immune response enables unimpeded growth and that the KA cells rapidly undergo the finite number of cell divisions of which they are capable, and then die without reaching immortality. © 1999 Cancer Research Campaign


*British Journal of Cancer* (1999) **80**(10), 1501–1505
© 1999 Cancer Research Campaign
Article no. bjoc.1999.0552

### Keratoacanthomas have an immunosuppressive cytokine environment of increased IL-10 and decreased GM-CSF compared to squamous cell carcinomas

**MA Lowes[1], GA Bishop[2], BE Cooke[3], RStC Barnetson[1] and GM Halliday[1]**

[1]Department of Medicine (Dermatology), University of Sydney at Royal Prince Alfred Hospital, Room W210, Blackburn Building D06, Sydney, NSW 2006, Australia; [2]Liver Immunobiology Unit, Centenary Institute of Cancer Medicine and Cell Biology, Sydney, NSW, Australia; [3]Anatomical Pathology, Prince of Wales Hospital, Sydney, NSW, Australia

**Summary** To investigate the relationship between keratoacanthoma (KA) and squamous cell carcinoma (SCC), cytokine mRNA in 12 KA and eight SCC were compared. Normal skin was also studied. Reverse transcription polymerase chain reaction (RT-PCR) was used to quantitate mRNA in each sample utilizing DNA standards. Glyceraldehyde-3-phosphate dehydrogenase (GAPDH) was used as an internal control, and CD3δ as an indication of the T-cell infiltrate. KAs showed a significant increase in interleukin (IL)-10, and a decrease in granulocyte macrophage colony-stimulating factor (GM-CSF) mRNA compared to SCCs. CD3δ mRNA was also increased in the KAs. There was no difference between KAs and SCCs in expression of lymphotoxin-α, IL-2, interferon-γ (IFN-γ), IL-13, transforming growth factor-β (TGF-β), or the pro-inflammatory cytokines IL-8 or tumour necrosis factor-α (TNF-α). These results indicate that KAs spontaneously resolve in an immunosuppressive environment. KAs grow rapidly over a period of weeks and then involute. It is possible that a suppressed immune response enables unimpeded growth and that the KA cells rapidly undergo the finite number of cell divisions of which they are capable, and then die without reaching immortality.

**Keywords:** skin cancer; tumour regression; tumour immunology; tumour development

The natural history of keratoacanthomas (KAs) is rapid growth followed by spontaneous involution, rarely invading or metastasizing, while squamous cell carcinomas (SCCs) show a variably progressive behaviour with local invasion and metastatic spread. There are also classical histological features distinguishing both these tumours. The process of KA growth and regression can be histologically documented. Mature KAs tend to have a characteristic architecture of a crateriform keratin-filled tumour, with the epidermis overhanging the sides forming a 'buttress', while SCCs show variable differentiation, anaplasia, pleomorphism, dyskeratosis and abnormal mitoses (Schwartz and Stoll, 1993; Schwartz, 1994). However, it is not possible to distinguish between KA and SCC on the basis of any one single clinical or histological feature (Schwartz, 1994), and misdiagnosis between these tumours is not uncommon. There are patterns of expression of various histopathological features, including ultrastructural appearance, cellular antigen and differentiation marker expression, and DNA studies, which can aid in accurate diagnosis.

The relationship between KAs and SCCs is still controversial. Suggestions include distinct entities, KAs may be a benign tumour in a spectrum with the more aggressive SCC at the malignant end, or KAs may represent a 'self-healing' or regressing form of SCC (Le Boit, 1995), much like actinic keratoses which also frequently regress spontaneously (Marks et al, 1986). The mechanism of KA resolution has also not been determined. Proposals include natural involution mirroring the hair cycle (Ghadially and Ghadially, 1993), rapid terminal differentiation secondary to immune stimulation (Prehn, 1996), or an activated immunological response directly destroying the tumour (Patel et al, 1994). Genetic events may also play a role in spontaneous KA resolution.

A recent study found the following features of regression were increased in KAs compared to SCCs: presence and degree of apoptosis, presence and degree of tumour degeneration, degree of stromal inflammatory infiltrate, presence of peritumoural neovascularization, and the presence of peritumoural fibrosis (Patel et al, 1994). The intratumoural inflammatory infiltrate in KAs consisted of significantly greater numbers of CD4+ T-cells, and increased markers of activation on tumour cells (CD36 and ICAM-1) and T-cells (interleukin (IL)-2 receptor, IL-2R). CD4+ T-cells have been similarly implicated in regression of melanomas (Tefany et al, 1991) and basal cell carcinomas (BCCs) (Hunt et al, 1994).

In this study, the cytokine profile of 12 KAs and eight SCCs was determined to provide an insight into the possible mechanism of spontaneous regression of KAs. The results indicate that the KAs resolve in an immunosuppressive cytokine microenvironment.

### MATERIALS AND METHODS

### Tumour collection

Lesions suspected of being a KA or SCC were collected from February 1994 to February 1996, from the Dermatology Outpatient Department and plastic surgery inpatients at Royal Prince Alfred Hospital. Specimens were also collected from

*Received 20 October 1998*
*Revised 2 February 1999*
*Accepted 9 February 1999*

*Correspondence to:* GM Halliday private dermatologists' rooms. Tumours were not collected for this study if the lesion was recurrent, or if the patient was an organ transplantation recipient, immunosuppressed, or had a history of prior chemotherapy. Ulcerated lesions were not included unless the tumour was large and the area of ulceration could be avoided. In all cases, informed consent was obtained as per the guidelines of the Central Sydney Area Health Service Human Ethics Committee. A 3–6 mm punch biopsy was obtained from the lesion as soon as possible after surgery, snap-frozen, and stored in liquid nitrogen until required. The remainder of the tumour was formalin-fixed and paraffin-embedded for routine histological examination.

### Normal skin collection

Samples of normal human skin were obtained during cosmetic breast reduction surgery, from healthy volunteers, or from the ends of ellipses during removal of clinically benign naevi at Royal Prince Alfred Hospital.

### Classification of KAs and SCCs

Paraffin sections were examined to classify the tumours as KA or SCC based on the histological criteria (Schwartz and Stoll, 1993; Schwartz, 1994). KAs are symmetrical tumours with a characteristic keratin-filled crater with a buttress, showing proliferation at the sides and base only. There are few mitotic figures at the base, and large homogeneous eosinophilic glassy cells, with minimal atypia. SCCs are evident as columns or clumps of tumour cells invading the dermis, and proliferation is seen throughout the lesion. There are frequent abnormal mitoses, varying degrees of differentiation and the cells may be anaplastic and pleomorphic. There may be single cell keratosis, horn pearl formation and dyskeratosis.

### Total RNA extraction and complementary DNA (cDNA) synthesis

Total RNA was prepared by acid guanidinium thiocyanate–phenol–chloroform extraction (Chomczynski and Sacchi, 1987) as previously described (Lowes et al, 1997). One microgram of biopsy RNA was reverse transcribed using Superscript RNAase $H^-$ enzyme kit (Gibco BRL, Gaithersberg, MD, USA) with Oligo dT (Boehringer, Sydney, NSW, Australia). A negative control consisted of a cDNA synthesis reaction omitting the reverse transcriptase enzyme (minus RT control). cDNA synthesis was performed in duplicate, termed cDNA1 and cDNA2.

### Non-competitive quantitative PCR

Polymerase chain reactions (PCR) were performed as previously described (Lowes et al, 1997). Each primer pair was optimized for magnesium chloride and *Taq* DNA polymerase concentrations and PCR amplification conditions. The PCR quantitation method of Dallman et al (1991) was modified to include external cytokine standards. Aliquots of 4.5 μl were removed from the samples and standards at each cycle, for most cytokines from cycle 28 onward. Aliquots were transferred to Hybond $N^+$ nylon membrane (Amersham, Little Chalfont, UK) using a dot-blot vacuum apparatus (Bio-Rad, Richmond, CA, USA) and membranes hybridized with a ATP-γ-$^{33}$P (DuPont, deNemours, France) end-labelled hybridization primer. Washed membranes were exposed to a phosphor imaging plate (Fuji Photo Film, Tokyo, Japan) and scanned on a Fujix Bio-imaging analyser BAS 1000 (Fujix, Tokyo, Japan) and the radioactivity in each dot was integrated. The number of molecules of cytokine cDNA for each specimen was calculated by the use of a standard curve prepared from the known number of molecules included in each run, as previously described (Bishop et al, 1997; Lowes et al, 1997). The reproducibility of the RT-PCR reaction was assessed by correlation of the results for cDNA1 and cDNA2 by linear regression analysis.

These results were averaged to give a final estimate of the number of molecules of cytokine cDNA. This was divided by the number of molecules of the internal control, glyceraldehyde-3-phosphate dehydrogenase (GAPDH), for the sample and multiplied by $10^6$.

The oligonucleotide cytokine amplification and hybridization primers used for GAPPH, IL-2, IL-8, IL-10, interferon (IFN)-γ, tumour necrosis factor (TNF)-α, lymphotoxin-α, and transforming growth factor (TGF)-β1 have been published (Lowes et al, 1997), as have granulocyte-macrophage colony-stimulating factor (GM-CSF) (Ehlers and Smith, 1991) and CD3δ (Yamamura et al, 1991).

### Statistical analysis

Non-parametric tests were used to test for significant differences as the data were not normally distributed. The Spearman rank correlation coefficient was used for linear regression analysis of cDNA syntheses, and the Mann–Whitney *U*-test used for tumour group analysis. A *P*-value of <0.05 was regarded as significant.

### RESULTS

### Characteristics of KAs and SCCs used for RT-PCR

Patients from whom tumours were collected were all over 50 years of age, and the average age (± standard deviation) of patients from both groups was very similar, 73.8 (±13.9) years for KAs and 73.4 (±11.6) years for SCCs. The sex distribution was equal in the KA group, and SCCs were collected from more females than males (M:F, 3:5). The average time to presentation in the KA group (8.8 ± 6.9 weeks) was statistically lower than the SCC group (13.6 ± 19.6 months) (Mann–Whitney *U*-test, $P < 0.05$). Tumours were predominantly located on sun-exposed sites: none were on the trunk and the only exception was an SCC located on the thigh. The majority of KAs were on the upper limbs (66.7%), while the majority of the SCCs were on the head and neck (62.5%).

### Cytokine mRNA analysis cDNA products were of predicted size by ethidium bromide-stained gel electrophoresis. Minus RT controls were negative for all cytokines. The quantitative method described here showed good reproducibility, with high correlations between cDNA 1 and cDNA 2 ($P < 0.0001$–0.05) for all cytokines.

### GAPDH

GAPDH was used as a positive internal control to correct for variations between specimens in the amount, quality and stability of mRNA. There was no statistical difference in the number of

 © 1999 Cancer Research Campaign

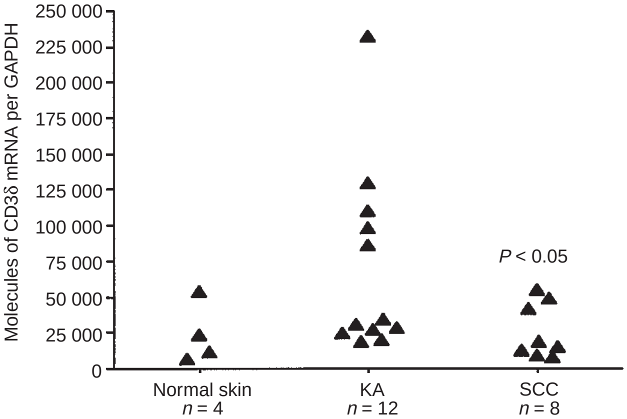

**Figure 1** CD3δ mRNA was elevated in KAs compared to SCCs. Ratio of number of molecules of CD3δ mRNA per $10^6$ molecules GAPDH mRNA. KA was significantly different to SCC, $P < 0.05$. KA compared to normal skin was not significantly different (Mann–Whitney *U*-test). Each point represents a single sample

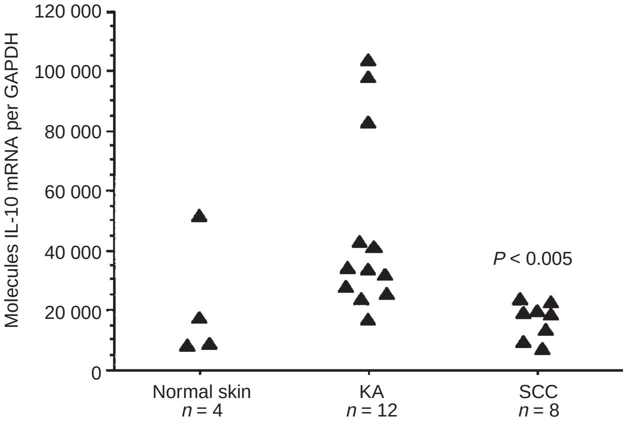

**Figure 2** IL-10 mRNA was elevated in KAs compared to SCCs. Ratio of number of molecules of IL-10 mRNA per $10^6$ molecules GAPDH mRNA. KA was significantly different to SCC, $P < 0.005$. KA compared to normal skin was not significantly different (Mann–Whitney *U*-test). Each point represents a single sample molecules of GAPDH between the tumour groups, or between the KAs and normal skin. The KAs contained a median number of molecules of GAPDH of $5.89 \times 10^5$ (range $1.14 \times 10^5$–$2.39 \times 10^6$), the SCCs had a median of $2.51 \times 10^6$ (range $3.10 \times 10^5$–$4.63 \times 10^6$), and normal skin had a median $4.60 \times 10^5$ (range $1.34 \times 10^5$–$1.23 \times 10^6$).

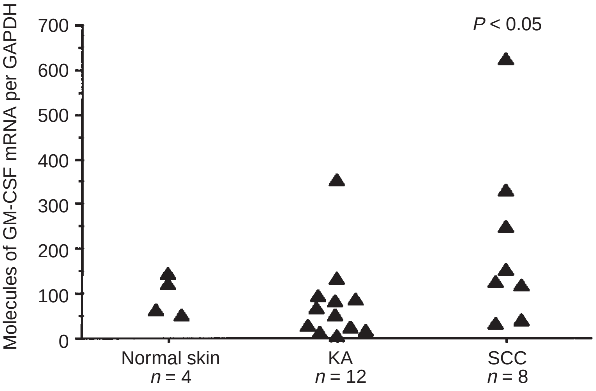

**Figure 3** GM-CSF mRNA was reduced in KAs compared to SCCs. Ratio of number of molecules of GM-CSF mRNA per $10^6$ molecules GAPDH mRNA. KA was significantly different to SCC, $P < 0.05$. KA compared to normal skin was not significantly different (Mann–Whitney *U*-test). Each point represents a single sample

### CD3δ

The ratio of the number of molecules of CD3δ mRNA per $10^6$ molecules of GAPDH mRNA is shown in Figure 1. KAs contained a median of $3.09 \times 10^4$ (range $1.71 \times 10^4$–$2.31 \times 10^5$), which was significantly higher than the SCCs which had a median of $1.55 \times 10^4$ (range $6.24 \times 10^3$–$5.32 \times 10^4$) ($P < 0.05$), but not significantly different from the normal skin which had a median of $1.58 \times 10^4$ (range $4.81 \times 10^3$–$5.24 \times 10^4$).

### IL-10 was elevated in KAs

The ratio of the number of molecules of IL-10 mRNA per $10^6$ molecules of GAPDH mRNA is shown in Figure 2. The median for IL-10 in the KAs was $3.33 \times 10^4$ (range $1.61 \times 10^4$–$1.03 \times 10^5$), which was significantly elevated in comparison to the SCCs, which had a median of $1.83 \times 10^4$ (range $6.42 \times 10^3$–$2.30 \times 10^4$) ($P < 0.005$). The median number of molecules of IL-10 in normal skin was $1.24 \times 10^4$ (range $7.27 \times 10^3$–$5.11 \times 10^4$) which was not significantly different to the KA group.

### GM-CSF was reduced in KAs

The ratio of the number of molecules of GM-CSF mRNA per $10^6$ molecules of GAPDH mRNA is shown in Figure 3. GM-CSF/GAPDH was significantly less in the KAs which had

**Table 1** Cytokines and growth factors which did not differ between KAs and SCCs

| Cytokine[a] | KA[b] | SCC[b] | Normal skin[b] |
|---|---|---|---|
| IL-2 | 58 (12–99) | 70 (11–132) | 76 (36–86) |
| LT-α | 539 (125–1.06 × $10^3$) | 693 (110–899) | 935 (356–2.02 × $10^3$) |
| IFN-γ | 3.19 × $10^4$ (4.71 × $10^3$–4.66 × $10^5$) | 2.42 × $10^4$ (1.18 × $10^4$–4.57 × $10^4$) | 8.39 × $10^3$ (3.67 × $10^3$–7.51 × $10^4$) |
| IL-13 | 4 (0–20) | 3 (0–13) | 19 (0–102) |
| IL-8 | 4.67 × $10^3$ (1.53 × $10^3$–1.12 × $10^5$) | 3.82 × $10^3$ (1.54 × $10^3$–9.37 × $10^3$) | 1.03 × $10^3$ (119–8.23 × $10^3$) |
| TNF-α | 80 (0–1.74 × $10^3$) | 69 (25–103) | 168 (68–591) |
| TGF-β | 695 (0–1.75 × $10^4$) | 1.31 × $10^3$ (304–6.38 × $10^3$) | 310 (0–1.04 × $10^3$) |

[a]No significant differences between KAs and SCCs for any cytokine, or between KAs and normal skin (Mann–Whitney *U*-test). [b]Median ratio of the number of molecules of cytokine mRNA per $10^6$ molecules of GAPDH (range) in KAs ($n = 12$), SCCs ($n = 8$) and normal skin ($n = 4$).

*© 1999 Cancer Research Campaign*

a median of 54 (range 0–348), compared to the SCC group which had a median of 133 (range 26–618) ($P < 0.05$). Normal skin had a median of 87 (range 46–141) for GM-CSF which was not significantly different to the KAs.

### Cytokines and growth factors which did not show a difference between KAs and SCCs

None of the other cytokines or growth factors investigated, lymphotoxin-α, IL-2, IFN-γ, IL-13, TGF-β, IL-8, or TNF-α showed significant differences between the KAs and SCCs, or between KAs and normal skin (Table 1).

### Correlation of cytokine mRNA with CD3δ mRNA

There were positive Spearman rank correlations between the GAPDH-corrected number of molecules of CD3δ for each tumour and IL-10 ($P < 0.005$), IFN-γ ($P < 0.0005$), IL-8 ($P < 0.05$), and TNF-α ($P < 0.001$). IL-13 showed a negative correlation with CD3δ ($P < 0.05$). The results for GM-CSF, the other Th1 cytokines, and TGF-β1 did not show a significant correlation with CD3δ mRNA.

### DISCUSSION

The cytokine mRNA profiles in 12 KA and eight SCC were examined to gain insights into the mechanisms underlying regression or malignancy of these two tumours respectively. Using a quantitative non-competitive RT-PCR, comparison of the cytokine mRNA profile of these two tumours has shown an increase in CD3δ and IL-10, and a decrease in GM-CSF, in the KA group compared to SCCs. The other cytokines analysed did not reveal any significant differences; these included lymphotoxin-α, IL-2, IFN-γ, IL-13, TGF-β, IL-8 and TNF-α.

CD3δ is a component of CD3, which is closely associated with the T-cell receptor, and its expression was used as a means to quantitate T-cells in the biopsies. The median result for CD3δ in KAs was significantly greater than in the SCC group and the magnitude of this difference was approximately twofold. This is similar to the magnitude of the difference of intratumoural CD3-positive cells detected immunohistochemically in a group of KAs compared to SCCs, where the mean number of CD3-positive cells per $mm^2$ was 78 for KAs and 33 for SCCs (Patel et al, 1994).

The present study found a significant increase in IL-10 in the KA group compared to the SCCs. This cytokine is usually considered to be immunosuppressive (Mosmann, 1994), and an important factor in the drive toward Th2 type immune responses (Enk et al, 1993). SCCs have shown greater IL-10 mRNA than seborrhoeic keratoses, a benign cutaneous tumour (Kim et al, 1995). Four BCC biopsies collected after completion of a course of IFN-α immunotherapy showed a dramatic decrease in IL-10 mRNA. However, in an animal sponge model of concomitant tumour immunity (EMT6 mammary tumour), the IL-10 mRNA was higher in rejecting lesions compared to the progressing tumours (Kurt et al, 1995). In murine models, IL-10 is capable of inducing effective anti-tumoural immune responses in vivo and inhibiting metastatic growth (Kundu et al, 1996).

GM-CSF has proven anti-tumour activity in mice (Dranoff et al, 1993) and man (Si et al, 1996). The KA group in this study showed less GM-CSF mRNA than the SCCs. GM-CSF has been shown to be reduced in other benign compared to malignant skin tumours (Yamamura et al, 1993). The low level of GM-CSF in the KAs may impair immune responses to these rapidly proliferating tumour cells. The absence of differences in the other T-cell or pro-inflammatory cytokines is also consistent with the conclusion that the immune system is not very active in the KA tumour.

KAs are very well-differentiated tumours; evidence includes their clinical, histological and ultrastructural features, and staining patterns for markers of differentiation. But, although the KA tumour cells are rapidly proliferating, they do so in a controlled and finite manner, and are not immortal. The cytokine peritumoural microenvironment described in KAs in this study, increased IL-10 and decreased GM-CSF, is consistent with an immunosuppressive cytokine milieu. This could impede the development of immune responses to these tumour cells, so that their growth is not limited by anti-tumour immunity. In this situation the cells would rapidly proliferate, but because they do not possess clonal immortality, have a finite number of proliferations before inevitable death.

This data supports the concept that KAs are a unique premalignant tumour of epidermal cell origin. However, perhaps if there is a brisk immune response to the KA tumour cells, this may promote development towards malignancy. There is evidence that the immune response can aid tumour progression. For example, macrophages may supply growth factors or activators of angiogenesis and proteolytic enzymes (Mantovani, 1994). Prehn (1996) has suggested that, in KAs, the immune infiltrate may be initially immunostimulatory. These stimulated tumour cells may tend to terminally differentiate, or grow rapidly before they have achieved immortality, or the immune infiltrate may change so that it is no longer stimulatory and the immunodependent KA regresses. The KAs analysed in the present study were from all phases of tumour development (proliferative, mature and involuting KAs), and it would be interesting to determine the nature of the cytokine environment in these different stages. It is possible that IL-10 may be primarily elevated during the proliferative phase of growth.

It is interesting to compare the results of the present study with our recently reported analysis of the cytokine mRNA profile of spontaneously regressing primary melanomas. These tumours showed a Th1 pattern (increased IL-2 and lymphotoxin-α) in comparison to non-regressing primary melanomas (Lowes et al, 1997). This profile suggests melanoma regression may be mediated by secretion of Th1 cytokines, which is in contrast with the pattern of expression discussed above for KAs. Thus spontaneous regression of cutaneous tumours may occur by different mechanisms, depending on many factors, including the inherent tumour properties, tumour antigenicity and peritumoural cytokine microenvironment.

### ACKNOWLEDGEMENTS

We are grateful to the dermatologists and plastic surgeons at Royal Prince Alfred Hospital for giving us access to their patients and providing biopsy samples. MAL is the recipient of a Postgraduate Faculty of Medicine Scholarship from the University of Sydney.

#### REFERENCES

Bishop GA, Rokahr KL, Lowes M, McGuinness PH, Napoli J, DeCruz DJ, Wong W-Y and McCaughan GW (1997) Quantitative reverse transcriptase-PCR amplification of cytokine mRNA in liver biopsy specimens using a non-competitive method. *Immunol Cell Biol* **75**: 142–147

*© 1999 Cancer Research Campaign*

Chomczynski P and Sacchi N (1987) Single-step method of RNA isolation by acid guanidinium thiocyanate-phenol-chloroform extraction. *Analyt Biochem* **162**: 156–159

Dallman MJ, Shiho O, Page TH, Wood KJ and Morris PJ (1991) Peripheral tolerance to alloantigen results from altered regulation of the interleukin 2 pathway. *J Exp Med* **173**: 79–87

Dranoff G, Jaffee E, Lazenby A, Golumbek P, Levitsky H, Brose K, Jackson V, Hamada H, Pardoll D and Mulligan RC (1993) Vaccination with irradiated tumor cells engineered to secrete murine granulocyte-macrophage colony-stimulating factor stimulates potent, specific, and long-lasting anti-tumor immunity. *Proc Natl Acad Sci USA* **90**: 3539–3543

Ehlers S and Smith KA (1991) Differentiation of lymphokine gene expression: the in vitro acquisition of T cell memory. *J Exp Med* **173**: 25–36

Enk AH, Angeloni VL, Udey MC and Katz SI (1993) Inhibition of Langerhans cell antigen-presenting function by IL-10. *J Immunol* **151**: 2390–2398

Ghadially R and Ghadially FN (1993). Keratoacanthoma. In: *Dermatology in General Medicine*, Fitzpatrick TB, Eisen AZ, Wolff K, Freeberg IM and Austen KF (eds), pp. 848–855. McGraw-Hill: New York

Hunt MJ, Halliday GM, Weedon D, Cooke BE and Barnetson RStC (1994) Regression in basal cell carcinoma: an immunohistochemical analysis. *Br J Dermatol* **130**: 1–8

Kim J, Modlin RL, Moy RL, Dubinett SM, McHugh T, Nickoloff BJ and Uyemura K (1995) IL-10 production in cutaneous basal and squamous cell carcinomas. *J Immunol* **155**: 2240–2247

Kundu N, Beaty TL, Jackson MJ and Fulton AM (1996) Antimetastatic and antitumor activities of interleukin 10 in a murine model of breast cancer. *J Natl Cancer Inst* **88**: 536–541

Kurt RA, Park JA, Panelli MC, Schluter SF, Marchalonis JJ, Carolus B and Akporiaye ET (1995) T lymphocytes infiltrating sites of tumor rejection and progression display identical Vβ usage but different cytotoxic activities. *J Immunol* **154**: 3969–3974

Le Boit PE (1995) Is keratoacanthoma a variant of squamous cell carcinoma. New insights into an old controversy … soon? *Am J Dermatopathol* **17**: 319–320

Lowes MA, Bishop GA, Crotty K, Barnetson RStC and Halliday GM (1997) T helper 1 cytokine mRNA is increased in spontaneously regressing primary melanomas. *J Invest Dermatol* **108**: 914–919

Mantovani A (1994) Tumor-associated macrophages in neoplastic progression: a paradigm for the in vivo function of chemokines. *Lab Invest* **71**: 5–16

Marks R, Foley P, Goodman G, Hage BH and Selwood TS (1986) Spontaneous remission of solar keratoses: the case for conservative management. *Br J Dermatol* **115**: 649–655

Mosmann TR (1994) Properties and functions of interleukin-10. *Adv Immunol* **56**: 1–26

Patel A, Halliday GM, Cooke BE and Barnetson RStC (1994) Evidence that regression in keratoacanthoma is immunologically mediated: a comparison with squamous cell carcinoma. *Br J Dermatol* **131**: 789–798

Prehn RT (1996) The paradoxical association of regression with a poor prognosis in melanoma contrasted with a good prognosis in keratoacanthoma. *Cancer Res* **56**: 937–940

Schwartz RA (1994) Keratoacanthoma. *J Am Acad Dermatol* **30**: 1–19

Schwartz RA and Stoll HL (1993) Squamous cell carcinoma. In: *Dermatology in General Medicine*, Fitzpatrick TB, Eisen AZ, Wolff K, Freeberg IM and Austen KF (eds), pp. 821–839. McGraw-Hill: New York

Si Z, Hersey P and Coates AS (1996) Clinical responses and lymphoid infiltrates in metastatic melanoma following treatment with intralesional GM-CSF. *Mel Res* **6**: 247–255

Tefany FJ, Barnetson RStC, Halliday GM, McCarthy SW and McCarthy WH (1991) Immunocytochemical analysis of the cellular infiltrate in primary regressing and non-regressing malignant melanoma. *J Invest Dermatol* **97**: 197–202

Yamamura M, Modlin RL, Ohmen JD and Moy RL (1993) Local expression of anti-inflammatory cytokines in cancer. *J Clin Invest* **91**: 1005–1010

Yamamura M, Uyemura K, Deans RJ, Weinberg K, Rea TH, Bloom BR and Modlin RL (1991) Defining protective responses to pathogens: cytokine profiles in leprosy lesions. *Science* **254**: 277–279

© 1999 Cancer Research Campaign
